# Breaking the membrane heredity paradox through de novo protocell formation

**DOI:** 10.1038/s41467-026-73667-z

**Published:** 2026-05-28

**Authors:** Satyam Khanal, Alessandro Fracassi, Alexander Harjung, Michael D. Burkart, Neal K. Devaraj

**Affiliations:** Department of Chemistry and Biochemistry, University of California, San Diego, CA USA

**Keywords:** Membrane lipids, Origin of life

## Abstract

Lipid membranes define cell boundaries, acting as gatekeepers for transport and signaling. A central paradigm in biology is that all cellular membranes descend from a common ancestral membrane, as they cannot be generated in the absence of pre-existing lipid structures. It is thus unclear whether lipid membranes can arise from membrane-less precursors. Here we demonstrate the de novo generation of lipid bilayers in the absence of any pre-existing membranes, membrane-bound proteins, or lipid nanostructure templates. Using acetate and cysteine as simple metabolites, lipid tails are constructed by soluble enzymes and spontaneously form diacyl lipids that assemble into vesicles. Pore-forming peptides facilitate precursor transport into vesicles, allowing the continuous generation of new lipids. Formation of glycolipid membranes creates compartments that can maintain proton gradients. Our findings demonstrate that lipid compartments can form without pre-existing membranes, establishing a unique route linking lipid synthesis to compartment formation and function.

## Abstract (web summary)

A central paradigm in biology is that cellular membranes descend from a common ancestral membrane, and it is currently unclear whether lipid membranes can arise from membrane-less precursors. In this study, the authors demonstrate the de novo generation of lipid bilayers in the absence of any pre-existing membranes, membrane-bound proteins, or lipid nanostructure templates.

## Introduction

**Fig. 1:**
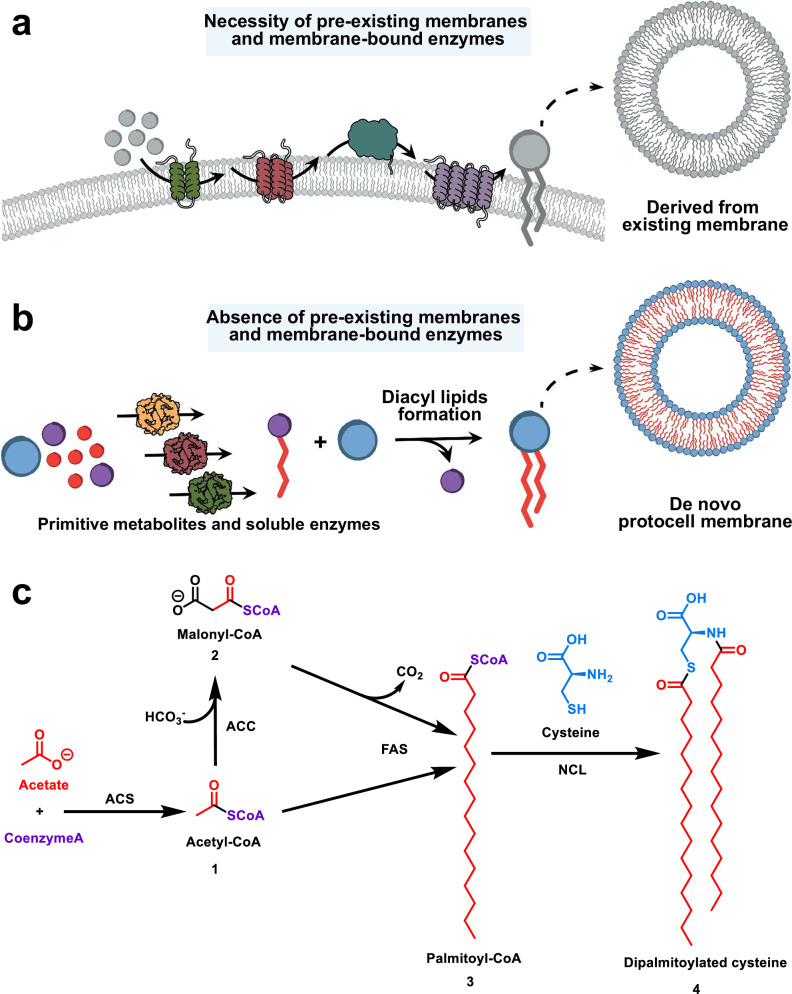
Schematic representation of de novo membrane formation driven by lipid synthesis from primitive metabolites. **a** Representative illustration of a generalized lipid biosynthesis process leading to the formation of new lipid membranes. The process depends on the presence of pre-existing membranes and membrane-bound proteins necessary for lipid synthesis. **b** Proposed approach for the formation of de novo generated membranes. Soluble primitive metabolites are converted into reactive single-chain amphiphiles by soluble enzymes, which react with primitive headgroup precursors to produce diacylated lipids that self-assemble into membranes. Membranes are formed in the absence of any pre-existing membranes, long-chain hydrocarbon lipids, or transmembrane proteins. **c** Reaction scheme for the synthesis of lipid 4. Three enzymes, acetyl-CoA synthetase (ACS), acetyl-CoA carboxylase (ACC), and fatty acid synthase (FAS) mediate the formation of palmitoyl-CoA 3 using acetate as a carbon source. Palmitoyl-CoA chemoselectively diacylates cysteine through native chemical ligation (NCL) to generate dipalmitoylated cysteine lipid 4, which is capable of spontaneous self-assembly, forming bilayer vesicles.

Cells compartmentalize themselves from their environment through bilayer membranes composed primarily of polar lipids such as phospholipids and glycolipids. Membrane lipid biosynthesis requires the action of several membrane-bound enzymes, making the process dependent on the existence of pre-existing lipid bilayers (Fig. 1a)^1^. This dependence suggests that membranes, like genetic material, are inherited and can be traced back to a common ancestral membrane^2,3^. Membrane heredity likely evolved as a necessity to preserve compartmentalization and prevent the loss of metabolites during cell division. If membranes had to be synthesized de novo at each division, there would be no reliable mechanism to ensure that cellular contents remained enclosed within a defined boundary. However, while membrane heredity is essential for life to propagate, it raises the paradox of how the first membranes emerged in the absence of pre-existing bilayers. Although membrane-like structures have been observed in complex abiotic organic mixtures^3^, lipid membrane formation from simple soluble precursors coupled to sustained lipid synthesis remains an open challenge. Several reports have demonstrated activity from reconstituting lipid membrane biogenesis pathways in artificial cells, but the lipid yields are typically low, and de novo membrane formation is not feasible, as the key enzymes in lipid biosynthesis are themselves membrane-bound^4–6^. Previous investigations have also demonstrated chemoenzymatic membrane formation from reactive surfactants, but these studies require the use of complex, long-chain hydrocarbon lipid precursors that form lipid nanoassemblies to template membrane formation^7,8^.

Here, we demonstrate the de novo formation of protocell membranes from soluble metabolites using a minimal set of soluble proteins. By generating acyl precursors using soluble enzymes and replacing the steps normally catalyzed by membrane-bound acyltransferases with chemical steps, simplified analogs of biological membrane lipids can be fully synthesized in the absence of pre-existing membranes (Fig. 1b). Acyl chains are enzymatically generated starting from acetate, a structurally simple two-carbon precursor and one of the five universal metabolites that can be formed abiotically through geochemical processes^9–11^. In situ-generated acyl chains spontaneously diacylate cysteine, an essential amino acid that has recently been shown to be important in prebiotic metabolism as a catalyst for peptide bond formation^12^. The diacylation products are polar lipids that resemble natural diacyl membrane lipids and spontaneously assemble to form protocell vesicles (Fig. 1c). Short pore-forming peptides can spontaneously insert into the de novo generated vesicles, forming channels that permit the transport of new precursors for continual membrane synthesis using entrapped proteins. Adapting the approach with more complex polar headgroups enables the formation of diacyl glycolipids, which are non-ionizable and self-assemble to form membranes that can maintain proton gradients, a conserved feature found in all life on Earth. Our biocatalytic strategy provides a route to bottom-up membrane construction in which reaction networks are used to generate amphiphiles from soluble molecules, with continuous input of carbon feedstocks sustaining lipid production.

## Results and discussion

### Palmitoyl-CoA synthesis from acetate

**Fig. 2:**
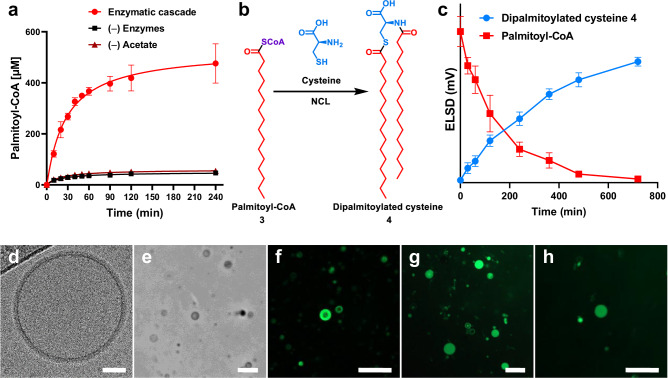
Enzymatic generation of palmitoyl-CoA from acetate, chemical synthesis of dipalmitoylated cysteine 4, and its spontaneous self-assembly into vesicles. **a** Palmitoyl-CoA synthesis from acetate mediated by the enzymatic cascade of acetyl-CoA synthetase (ACS), acetyl-CoA carboxylase (ACC), and fatty acid synthatse (FAS). Data points presented as means ± s.d. (*n* = 3 technically independent samples). **b** Native chemical ligation (NCL) reaction scheme of dipalmitoylated cysteine 4 synthesis from palmitoyl-CoA and cysteine in bicine buffer pH 8.5. **c** Formation of lipid 4 over 12 h (blue) and concomitant consumption of palmitoyl-CoA (red) in bicine buffer pH 8.5. Data points presented as means ± s.d. (*n* = 3 technically independent samples). **d** Cryo-EM image of a vesicle of lipid 4 generated in situ from the reaction between palmitoyl-CoA and cysteine. Scale bar = 20 nm. **e** Phase-contrast microscopy image of membrane-bound vesicles resulting from the synthesis of lipid 4 and its spontaneous self-assembly in the presence of 10 mol% cholesterol. Scale bar = 5 µm. **f** Fluorescence microscopy image of vesicles of 4 stained with 0.1 mol% BODIPY-FL. Scale bar = 5 µm. **g** Fluorescence microscopy image of vesicles of 4 showing encapsulation of HPTS in the aqueous core. Scale bar = 5 µm. **h** Fluorescence microscopy image of vesicles of 4 showing encapsulation of GFP in the aqueous core. Scale bar = 5 µm. Source data are provided as a Source Data file.

To form lipid membranes de novo from non-lipidic metabolites, the long-chain hydrocarbon tails that are essential for membrane assembly must be synthesized from structurally simple, soluble molecules. For this purpose, we selected well-characterized enzymes that function as soluble catalysts, providing a convenient experimental platform for generating amphiphilic lipid precursors in solution. Starting from acetate, we combined three enzymatic reactions to build the long-chain acyl-CoA thioester palmitoyl-CoA. We used *E. coli* acetyl-CoA synthetase (ACS) to convert acetate to acetyl-CoA^13^, human acetyl-CoA carboxylase (ACC) for producing malonyl-CoA from the generated acetyl-CoA^14,15^, and a type I fatty acid synthase from *Corynebacterium glutamicum* (FAS) to synthesize palmitoyl-CoA from acetyl-CoA and malonyl-CoA (Fig. 1c)^16^. Initially, we individually assessed the activity of ACS, ACC, and FAS, which provided yields of 66% for acetyl-CoA, 71% for malonyl-CoA, and 63% for palmitoyl-CoA, over 4 h (Supplementary Figs. 1–3). Combining the three enzymes provided palmitoyl-CoA with a 42% yield within 4 h (Fig. 2a and Supplementary Fig. 4). No palmitoyl-CoA was observed in control experiments lacking any of the three enzymes or in the absence of acetate (Fig. 2a).

### Diacylation of cysteine by palmitoyl-CoA

In living cells, long-chain acyl-CoA thioesters like palmitoyl-CoA are used to acylate hydrophilic head groups and generate polar lipids like phospholipids, sphingolipids, and glycolipids. The acylation reactions are catalyzed by proteins, which typically require integration into lipid membranes to be fully functional^17–19^. We previously demonstrated that thioesters can spontaneously acylate aminothiol functional groups by native chemical ligation (NCL)^20–22^. Recent work has observed that protocells can be generated by the spontaneous dual *N*- and *S*-acylation of cysteine^23^. However, pre-synthesized unnatural long-chain thioesters were required to template membrane formation, and true de novo synthesis was not demonstrated. We hypothesized that cysteine might be similarly diacylated in the presence of in situ-generated palmitoyl-CoA thioesters, forming a diacyl lipid capable of assembling into membranes. While cell membranes are often formed by diacylation of a glycerol backbone, it has recently been discovered that certain thermophilic bacteria form a large percentage of their membrane lipids by diacylation of a serine amino acid-derived backbone^24,25^. Amino acid diacylation may be a biochemically relevant mechanism for forming membrane lipids, and such lipids could have served as primordial precursors to cellular lipids.

We initially tested whether palmitoyl-CoA was capable of diacylating cysteine. We mixed 2 mM of cysteine with 4 mM palmitoyl-CoA at 37 °C in bicine buffer at pH 8.5 (Fig. 2b) and observed significant dipalmitoylated cysteine **4** formation by HPLC-ELSD-MS, along with the consumption of palmitoyl-CoA (Fig. 2c). A pH of 8.5 was selected to increase the fraction of cysteine present as thiolate, facilitating the first acylation step^26^. We confirmed synthesis of **4** by comparison with a chemically synthesized standard (Supplementary Fig. 5). After 8 h, we observed 1.6 ± 0.3 mM of lipid **4**, corresponding to 80% yield. Intriguingly, we observed only trace formation of the monoacylated lipid product, including at early time points. Even when using substoichiometric amounts of palmitoyl-CoA, we primarily observed formation of the diacylated compound **4** with minimal formation of monoacylated cysteine (Supplementary Fig. 5). Considering the low critical micelle concentration (cmc) reported for palmitoyl-CoA (35 µM at pH 8.5)^27^, we attribute our observation to the rate-limiting step being the first acylation. Once acylated, the lipid likely forms mixed micelles with palmitoyl-CoA, positioning another thioester in proximity to the reactive thiol, leading to a more rapid second acylation. These observations indicate that cooperative lipidation mechanisms can drive the formation of diacylated lipid species in the absence of membrane-bound enzymes.

### Cysteine lipids form protocell membranes

Microscopy studies were conducted to determine if dipalmitoylated-cysteine lipids could form membrane-bound vesicles. We observed small ( < 2 μm) lipid structures formed by self-assembly of **4** (Supplementary Fig. 6), which falls within a favorable protocell size range for supporting primitive biochemical processes^28^. Vesicle formation was confirmed by cryo-electron microscopy (cryo-EM) (Fig. 2d). The small size of the vesicles observed may be attributed to the fully saturated palmitoyl tails of lipid **4**, which restrict membrane fluidity, influencing vesicle assembly^29^. Indeed, the phase transition temperature of membranes of **4** was found to be 60.4 °C (Supplementary Fig. 6). However, due to the limitations of optical microscopy in visualizing submicron vesicles, we sought to obtain larger vesicles to facilitate experimental observation and characterization. Since sterols can disrupt the long-range lateral order of saturated lipids and help fluidize membranes, we hypothesized that addition of low concentrations of cholesterol (10-15 mol%) would support the assembly of larger vesicles from lipid **4**^30,31^. We found that addition of 10 mol% of cholesterol during the synthesis of **4** leads to the formation of vesicles between 1-5 µm in diameter that were easily observable by phase-contrast and fluorescence microscopy using 0.1 mol% BODIPY-FL (Fig. 2e, f and Supplementary Fig. 6). As a control, we verified that palmitoyl-CoA, cysteine, cholesterol, or any combination of these components did not lead to membrane formation (Supplementary Fig. 7). To further characterize the vesicle size distribution upon in situ formation of lipid **4**, we carried out dynamic light scattering (DLS) analyses in the absence and presence of cholesterol (Supplementary Fig. 8). In both conditions, the DLS profiles revealed highly polydisperse samples, consistent with the vesicle population spanning nanometer to micrometer length scales, in agreement with previous findings^32,33^. Vesicles formed from **4** were able to encapsulate small polar molecules such as 8-hydroxypyrene-1,3,6-trisulfonic acid (HPTS), a highly polar fluorescent dye (Fig. 2g). Similarly, synthesis of **4** in the presence of green fluorescent protein (GFP) also led to spontaneous entrapment of the protein, indicating that larger macromolecules can be encapsulated during vesicle formation (Fig. 2h). The ability to encapsulate proteins during de novo lipid vesicle formation suggests that early protocells may have been able to capture primitive metabolic networks in a confined environment to sustain biochemical activity and couple metabolism with compartmentalization^34,35^.

### Chemoenzymatic de novo membrane formation

**Fig. 3:**
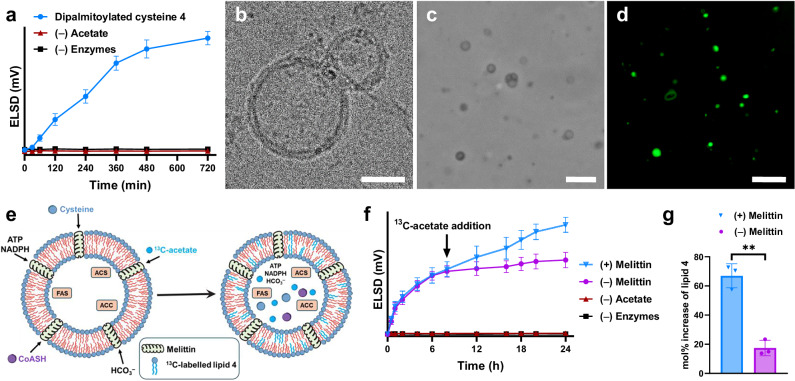
Chemoenzymatic synthesis of dipalmitoylated cysteine lipid 4 leading to de novo formation of vesicles. **a** Chemoenzymatic de novo generation of lipid **4** over 12 h. Negative controls show that no product is formed in the absence of acetate or without the enzymes acetyl-CoA synthetase (ACS), acetyl-CoA carboxylase (ACC), and fatty acid synthase (FAS). Data points presented as means ± s.d. (*n* = 3 technically independent samples). **b** Cryo-EM image of membrane-bound vesicles of lipid **4** generated de novo chemoenzymatically. Scale bar = 20 nm. **c** Phase-contrast microscopy image of membrane-bound vesicles of lipid **4** in the presence of 10 mol% cholesterol. Scale bar = 5 µm. **d** Spontaneous GFP encapsulation during the chemoenzymatic de novo formation of lipid **4** membranes in the presence of 10 mol% cholesterol. Scale bar = 5 µm. **e** Schematic representation of melittin-mediated pore formation in dipalmitoylated cysteine lipid **4** vesicles. New isotopically labeled lipid **4** (depicted in light blue) is synthesized after addition of fresh precursors that enter the vesicles via the melittin pores. **f** Chemoenzymatic de novo formation of lipid **4** monitored over time. 10 mol% melittin was added after 8 h of diacylation and incubated for 1 h. Fresh reaction precursors including ^13^C-acetate and cysteine were added and the reaction followed for a further 8 h. As shown in the graph, significant additional ^13^C-labeled lipid **4** is synthesized when melittin is present. Negative control without melittin addition showed reduced formation of lipid **4**. No lipid formation was observed in controls lacking acetate or enzymes. Data points presented as means ± s.d. (*n* = 3 technically independent samples). **g** Increase in the amount of lipid **4** after the addition of new reaction precursors in the presence or absence of melittin. Data points presented as means ± s.d. (*n* = 3 technically independent samples), and the significance determined using an unpaired t-test (two-tailed). ***P = 0.0009. Source data are provided as a Source Data file.

Since our goal was to generate lipid membranes from simple metabolic precursors using a minimal set of soluble enzymes in the absence of pre-existing membranes or lipid nanostructures, we next explored if the biosynthesis of palmitoyl-CoA **3** could be combined with chemical diacylation of cysteine for chemoenzymatic de novo membrane formation (Fig. 3). Optimization of the reaction conditions allowed both the enzymatic and chemical reactions to occur simultaneously. Briefly, cysteine was added to a reaction mixture containing acetate, and the three enzymes ACS, ACC and FAS with the necessary cofactors were incubated at 37 °C. The formation of lipid **4** was monitored by HPLC-ELSD-MS, reaching a yield of 43% after 12 h (Fig. 3a). Small lipid structures ( < 2μm) were detected by phase-contrast microscopy (Supplementary Fig. 9), and cryo-EM analysis revealed the formation of nanometer sized bilayer vesicles (Fig. 3b). Since the formation of palmitoyl-CoA **3** is considerably faster than the subsequent chemical diacylation of cysteine, we propose that vesicle formation may proceed through initial formation of compound **3** micelles, within which lipid **4** is generated, followed by transition to bilayer structures. Once bilayers are formed, expansion can proceed through transfer of palmitoyl-CoA from micelles into pre-existing membranes, where tighter packing might promote retention relative to micelles, followed by conversion of palmitoyl-CoA to lipid **4**. Consistent with this mechanism, generalized polarization (GP) measurements show a pronounced decrease upon addition of palmitoyl-CoA to hydrated vesicles of lipid **4**, suggesting intercalation into pre-existing membranes even when starting from fully formed vesicles (Supplementary Fig. 10). Addition of 10 mol% cholesterol (relative to the final concentration of lipid **4**) to the reaction mixture led to the formation of larger vesicles with diameters reaching up to 5 μm (Fig. 3c, d). As in the case of vesicles formed from chemically synthesized lipid **4** (Fig. 2g, h), de novo vesicles generated during the chemoenzymatic reaction were able to spontaneously encapsulate HPTS (Supplementary Fig. 9) and GFP (Fig. 3d) if these molecules were present in the buffer at the start of the reaction. The bilayer thickness for the de novo vesicles was estimated to be ~5 nm based on cryo-EM analysis (Supplementary Fig. 11).

Despite the presence of competing reactive species such as the acetyl-CoA and malonyl-CoA thioesters, cysteine underwent selective diacylation with palmitoyl-CoA to form **4**. No products bearing acetylated or malonylated tails were detected by HPLC-ELSD (Supplementary Fig. 12). One possible explanation is that the consumption of acetyl-CoA and malonyl-CoA by enzymes such as ACS, ACC, and FAS to produce palmitoyl-CoA occurs more rapidly compared to the much slower NCL-mediated diacylation reaction (Fig. 2a, c). We also found that the acylation of cysteine with amphiphilic palmitoyl-CoA is strongly favored over acylation with more polar thioesters such as acetyl-CoA and malonyl-CoA (Supplementary Fig. 12). This preference may be attributed to the partitioning of cysteine into palmitoyl-CoA micelles, thus contributing to the observed chemoselective acylation^36^.

### Peptide pores support lipid synthesis

We hypothesized that if the enzyme catalysts are entrapped within the de novo-formed vesicles, membrane synthesis could be sustained internally. However, to enable continued lipid synthesis, there would also need to be mechanisms by which small molecule precursors can reach the interior of the protocells without the use of transmembrane protein channels, which would have required pre-existing lipid membranes for proper insertion and folding. Melittin is a short 26-amino acid soluble lytic peptide from bee venom that induces concentration-dependent pore formation in lipid membranes^37–40^. At low concentrations, melittin associates with the surface of lipid membranes without compromising their integrity, but at higher concentrations it inserts into the membrane and induces channel formation^41^. In phospholipid membranes, the pore sizes formed by melittin are between 2.6 and 4.8 nm in diameter which lets small molecules, such as fluorescent dyes, to travel freely through the membranes while preventing the translocation of larger macromolecules such as enzymes^42^. We sought to investigate whether melittin could similarly interact with de novo-formed membranes composed of lipid **4**, generating pores that permit the influx of small molecules while retaining encapsulated enzymes within the vesicles. We prepared vesicles of **4** in reaction buffer containing HPTS and removed the excess unencapsulated dye via spin-filtration (Supplementary Fig. 13). We then tested dye leakage in response to increasing amounts of added melittin (0–25 mol%). Upon addition of 10 mol% melittin, vesicles lost their internal fluorescence within 1 hour of incubation, indicating membrane permeabilization, while retaining their morphology (Supplementary Fig. 13). In contrast, addition of 25 mol% melittin caused complete membrane disruption, as vesicles could no longer be visualized by microscopy, although lipid **4** was still present in the sample, as confirmed by HPLC-ELSD-MS analysis (Supplementary Fig. 14). As a control, in the absence of melittin, no leakage of HPTS was observed over a 16-hour period, supporting the role of melittin in membrane pore formation (Supplementary Fig. 15). Finally, analogous experiments with vesicles encapsulating GFP showed that the encapsulated protein was retained in the presence of 10 mol% melittin, indicating that melittin pores only allow the passage of small molecules across membranes while excluding larger macromolecules (Supplementary Fig. 15).

After demonstrating that short peptides can insert into the chemoenzymatically generated membranes and form nanochannels, we investigated if subsequent addition of reactive precursors would lead to additional lipid synthesis (Fig. 3e). Since macromolecules like GFP are encapsulated during de novo protocell formation and show no significant leakage after melittin treatment, we reasoned that the enzymes ACS, ACC, and FAS are likewise encapsulated and retained inside the newly formed lipid **4** vesicles (Fig. 3e). We chemoenzymatically formed **4** in the presence of HPTS, confirmed vesicle assembly by microscopy, and then removed non-encapsulated HPTS, enzymes, and other reactants by spin-filtration. We then added 10 mol% melittin to the vesicle population and incubated at 37 °C for 1 h. Following confirmation of pore formation, indicated by HPTS leakage, we supplemented the system with fresh reagents and cofactors, including cysteine, ^13^C-labeled acetate, CoASH, HCO_3_^–^, ATP, NADPH, and incubated for 8 h. Lipid formation was monitored by HPLC-ELSD, revealing the formation of new ^13^C-labeled lipids (Fig. 3f), corresponding to a 67 mol% increase in total lipid content (Fig. 3g). In contrast, in the absence of melittin, lipid levels increased by only 17 mol% over the same period (Fig. 3g). The significant difference in lipid synthesis with and without melittin is likely due to the limited permeability of the vesicle membrane, as the newly added reaction precursors would have difficulty entering the vesicles simply through passive diffusion. Consistent with prior studies, the amount of de novo synthesized lipid was insufficient to directly observe vesicle growth by optical microscopy^5^.

### Non-ionizable de novo membranes maintain proton gradients

**Fig. 4:**
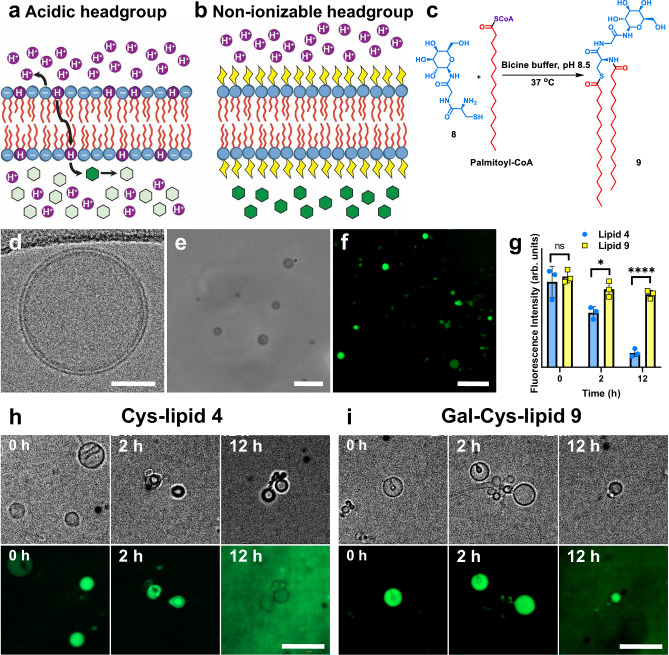
De novo formation of vesicles capable of maintaining a proton gradient. Representative illustrations of a proton gradient across membranes containing acidic **a** versus non-ionizable **b** lipid headgroups. Proton shuttling across the membrane is facilitated by protonation and flip-flop of the acidic lipid, while in membranes with non-ionizable headgroups, protons must cross the membrane in their charged form, which is energetically less favorable. **c** Reaction scheme for lipid **9** synthesis from palmitoyl-CoA and galacto-HG **8** in bicine buffer pH 8.5. **d** Cryo-EM image of a vesicle of lipid **9** generated from palmitoyl-CoA and **8** in the presence of 10 mol% cholesterol. Scale bar = 20 nm. **e** Phase-contrast microscopy image of de novo generated vesicles formed through the chemoenzymatic reaction using acetate and **8** as precursors, in the presence of 10 mol% cholesterol. Scale bar = 5 µm. **f** Fluorescence microscopy image of vesicles of **9** formed through the chemoenzymatic reaction showing encapsulation of GFP in the aqueous core. Scale bar = 5 µm. **g** Time-dependent decay of a pH gradient in vesicles composed of either lipid **4** or **9**. Background-subtracted fluorescence intensity of encapsulated HPTS was measured at 0 h, 2 h and 12 h after buffer exchange from bicine (pH = 8.5) to citrate (pH = 4.5). Data points are presented as means ± s.d. (*n* = 3 technically independent samples), and the significance determined using an unpaired t-test (two-tailed). 0 h, *P* = 0.6130; 2 h *P = 0.0143; 12 h ****P < 0.0001. (**h-i**) Brightfield (*top*) and fluorescence (*bottom*) microscopy images showing pH gradient decay in vesicles composed of lipid **4** (**h**) and galactolipid **9** (**i**) at 0 h, 2 h, and 12 h after buffer exchange. Scale bars = 5 µm. Source data are provided as a Source Data file.

While cysteine offers a highly simple polar substrate for diacylation and membrane lipid formation, cellular membrane lipids are more complex, often bearing sugars or phosphorylated polar head groups. To better mimic the structural features of natural lipids, we considered alternative polar head groups with aminothiol reactive groups that might also be diacylated to form more complex lipid structures. An essential function of lipid membranes in cells is their maintenance of proton gradients, a requirement for chemiosmosis^40^. However, vesicles formed from lipids containing carboxylic acid headgroups have difficulty supporting proton gradients due to proton shuttling across the membrane (Fig. 4a). The need for proton gradient control suggests that more complex polar lipids developed early during the emergence of life, bridging prebiotic chemistry and early protocells^43,44^. We speculated that introducing a non-ionizable sugar head group rather than a carboxylic acid head group could render the resulting lipid membranes capable of maintaining proton gradients (Fig. 4b).

To test this, we synthesized a short glycopeptide polar head group galactosaminothiol (galacto-HG) **8** (Supplementary Fig. 16) and showed that spontaneous diacylation and vesicle formation occur in the presence of palmitoyl-CoA to form non-canonical galactolipid **9** (Fig. 4c and Supplementary Fig. 17). Galacto-HG **8** undergoes diacylation during the enzymatic synthesis of palmitoyl-CoA to generate galactolipid **9**. Optimization of the reaction conditions enabled concurrent synthesis of enzymatically generated palmitoyl-CoA, diacylation of galacto-head group **8**, and vesicle formation (Fig. 4d, e). Vesicles formed from galactolipid **9** were shown to be able to encapsulate small molecules (Supplementary Fig. 18), and proteins (Fig. 4f), and they could support additional lipid synthesis in the presence of the pore forming peptide melittin, similar to vesicles formed from **4** (Supplementary Fig. 19).

To assess whether membranes formed from either lipid **4** or lipid **9** differ in their ability to maintain proton gradients, we generated vesicles de novo using cysteine or galacto-HG **8** as reactive head groups in the presence of HPTS in bicine buffer (pH 8.5) and subsequently triggered a proton gradient by exchanging the external buffer for citrate buffer (pH 4.6) (Supplementary Fig. 20). The HPTS dye exhibits high fluorescence in alkaline media, but its fluorescence is quenched by more than 99% in more acidic environments^45^. Following buffer exchange, fluorescence measurements indicated that a proton gradient was initially maintained across both types of membranes. However, the fluorescence intensity of the vesicles decreased over time in both cases, likely reflecting acidification of the internal lumen of the vesicles due to proton influx (Fig. 4g). In vesicles composed of dipalmitoylated-cysteine **4**, fluorescence decreased by 83 ± 6% over 12 h, whereas vesicles formed by galactolipid **9** showed only a 19 ± 7% decrease over the same time period (Fig. 4g). It is worth noting that, even though lipid **4** vesicles dissipate the proton gradient over time, they preserve it far better than fatty acid vesicles, which typically lose transmembrane pH gradient within seconds^43,44^. By comparison, the behavior of galactolipid **9** is consistent with that of tightly packed phospholipid membranes such as DPPC, which is used in liposome formulations to reduce membrane permeability and support prolonged retention of transmembrane ion and pH gradients^46^. Confocal microscopy images corroborated the fluorescence quenching observed for lipid **4** vesicles (Fig. 4h), while confirming the persistence of fluorescence in lipid **9** vesicles even after 12 h (Fig. 4i). Lipid **4** possesses a small, acidic polar head group, which may facilitate the shuttling of protons from the outer buffer to the inner lumen of the membranes after buffer exchange, similar to observations with single-chain fatty acids, albeit occurring at a slower rate due to the presence of two saturated and long-chain fatty-acyl hydrocarbon tails^47,48^. Indeed, control experiments performed using oleic acid vesicles showed a much more rapid drop in fluorescence upon buffer exchange (Supplementary Fig. 21). In contrast, galactolipid **9** contains a galactosamino moiety as the head group, which does not possess an easily ionizable functional group and is significantly larger in size. The ability to maintain and harness proton gradients could have conferred protocells with competitive advantages and driven the evolution of more complex membrane systems.

We demonstrate that protocell membrane formation can take place in the absence of pre-existing membranes or lipid templates, upending a long-standing dogma in cellular biology. Our study reveals how soluble enzymes and small-molecule metabolites can drive the formation of lipid membranes, establishing that functional membrane compartments can arise directly from soluble precursors. These findings define a minimal framework for membrane formation in which lipid synthesis, self-assembly, and compartment function are directly coupled through soluble reaction networks. In this context, membrane assembly emerges from the interplay of enzymatic lipid production and supramolecular organization, allowing compartment boundaries to form together with the chemical processes that sustain them. Controlling lipid unsaturation will be an important direction for future work, particularly for accessing bilayers with lower phase transition temperatures that better support functional studies. Our work also suggests alternative routes for encoding membrane formation in artificial cells and ways to generate lipid structures de novo for applications in nanotechnology, medicine, and materials science.

## Methods

### Enzymatic activity assays

Enzyme activities were assessed using both indirect (substrate consumption) and direct (product formation) methods. Indirect measurements for ACS and ACC were performed by quantifying AMP production using the AMP-Glo^TM^ assay kit (Promega). FAS activity was evaluated by monitoring NADPH consumption spectrophotometrically. Direct quantification of enzymatic activity was performed using an acetyl-CoA assay kit for ACS, a malonyl-CoA ELISA assay for ACC, and HPLC-ELSD detection of palmitoyl-CoA for FAS. Further methodological details are provided in the Supplementary Information.

### Chemoenzymatic membrane formation from acetate

Lipids were generated starting from acetate using ACS, ACC, and FAS and cysteine or compound **8** as the reactive head group. Briefly, 12.25 μL of 1 M bicine buffer, pH 8.5 (containing 100 mM TCEP), 1.75 μL of 200 mM acetate (in sterile H_2_O), 1.75 μL of 200 mM CoASH (in sterile H_2_O), 1.75 μL of 200 mM HCO_3_^-^(in sterile H2O), 3.75 μL of 200 mM ATP (in sterile H_2_O), 3.75 μL of 200 mM NADPH (in sterile H_2_O), 5 μL of ACS (50 μM in elution buffer), 5 μL of 50 μM ACC (in sterile H_2_O), 12.5 μL of FAS (20 µM in elution buffer), and 2.5 μL of 10 mM reactive head group were added to a glass vial and tumbled at 37 °C. The reaction progress was monitored over 12 h using HPLC-ELSD-MS. For reactions conducted in the presence of cholesterol (10 mol%), a thin film of cholesterol was prepared in a glass vial, dried under N_2_, and the chemoenzymatic lipid synthesis was then performed in the same vial at 37 °C.

### Encapsulation of HPTS in de novo-formed membranes

The de novo vesicle formation reactions of lipids **4** and **9** were carried out as described above, with the sole modification of adding 1 µL of 1 mM HPTS solution to the reaction mixture. When the reaction was completed, the mixture was transferred onto a Sephadex G-50 spin column and centrifuged for 4 min at 0.3 × g to remove unencapsulated dye. Encapsulation of HPTS was confirmed by confocal microscopy.

### HPTS leakage assay of de novo formed lipid vesicles after melittin incubation

To evaluate melittin-induced pore-formation, HPTS leakage was monitored after its encapsulation in vesicles of lipids **4** and **9**. Vesicle formation reactions and HPTS encapsulation were carried out as described above. Each sample was split into two equal aliquots, and fluorescence was recorded using a microplate reader. Melittin (10 mol%) was added to one aliquot, while an equivalent volume of water was added to the other aliquot as a negative control. Following a 1-h incubation at 37 °C, both samples were spin-filtered with a Sephadex G-50 SEC column at 0.3 × g for 4 min to remove released HPTS, after which fluorescence was measured again.

### ^13^C-labeled chemoenzymatic lipid synthesis

Vesicle formation reactions with lipid **4** and **9**, including HPTS encapsulation, were performed as described above. Melittin (10 mol% relative to lipid) was then added, and the suspensions were incubated at 37 °C for 30 min. Unencapsulated HPTS was removed by Sephadex G-50 spin-filtration (0.3 × g, 4 min). The filtrate was supplemented with reactive headgroup (250 μM), ^13^C-labeled acetate (10 mM), CoASH (10 mM), HCO_3_^–^ (7 mM), ATP (15 mM), and NADPH (15 mM), and the mixture was incubated for an additional 8 h at 37 °C. The formation of ^13^C-labeled lipids was quantified by HPLC-ELSD-MS.

### Proton gradient assay

Vesicles composed of lipid **4** and **9** and encapsulating HPTS were formed in bicine buffer (pH 8.5). Unencapsulated dye was removed by Sephadex G-50 spin-filtration (0.3 × g, 4 min). The bicine buffer (pH = 8.5) was then exchanged for citrate buffer (pH = 4.6) using the same spin-filtration protocol, after which HPTS fluorescence was monitored over time with a microplate reader.

### Statistics and reproducibility

All experiments presenting representative micrographs or images were performed at least three times independently, with consistent results obtained across replicates.

### Reporting summary

Further information on research design is available in the Nature Portfolio Reporting Summary linked to this article.

## Supplementary information

Supplementary Information

Reporting Summary

Transparent Peer Review file

## Source data

Source Data

## Data Availability

All data are available in the main text or Supplementary Information, and from the corresponding author(s) upon request. Source data are provided with this paper.
