# Supplementary material for: Breaking the membrane heredity paradox through de novo protocell formation
Source: Nat Commun. 2026 May 28;17:6920. doi: 10.1038/s41467-026-73667-z (PMC13388977; doi:10.1038/s41467-026-73667-z)
Supplement: Supplementary file 3 — Transparent Peer Review file [file 41467_2026_73667_MOESM3_ESM.pdf]

nature portfolio

Peer Review File

Breaking the membrane heredity paradox through de novo protocell formation

Corresponding Author: Professor Neal Devaraj

This file contains all reviewer reports in order by version, followed by all author rebuttals in order by version.

Version 0:

Reviewer comments:

Reviewer #1

(Remarks to the Author)
This article used protein enzymes to make palmityl-coA and then diacyl lipid from aqueous precursors, which is novel. Reactants are prebiotic using acetate and cysteine. The yields were good. The resulting protocells were then characterized for growth both with and without a general membrane pore (mellettin). The ability of these membranes to maintain a proton gradient was also determined.
The paper was straightforward and of interest to a wide audience, including syth bio, OOL, astrobio, and metabolic studies.

The approach was appropriate for the goals of the paper. They used multiple types of microscopy to confirm the morphology of their structures, and the encapsulation ability of the protocells. The kinetics were also measured in more than one way, and the paper presented very clear statistical analysis in figures and figure captions.

While the reactants were obliviously relevant to the goals of origins of life, the use of extant enzymes was not justified in the introduction. A more nuanced discussion of the role of highly evolved and cherry-picked enzymes/cofactors is warranted, especially if the authors want to claim this is important for evolution. Also Lines 38-40 state there is a gap in forming these amphiphiles, however there are lots of mechanisms for generating membrane forming materials abiotically (as demonstrated in meteorite samples, Deamer 1985) and therefore the need for this process could be late evolutionarily.
(1)
Deamer, D. W. Boundary Structures Are Formed by Organic-Components of the Murchison Carbonaceous Chondrite. Nature 1985, 317 (6040), 792–794.

Minor comments:
Positive controls for comparing growth in phospholipid membranes or proton permeation in phospholipid membranes would have been a good benchmark. (or comparison to literature values?)
I couldn't find the manufacturer for the Melittin.
Just an editing comment: punctuation is generally prior to ref superscripts

Reviewer #2

(Remarks to the Author)
I co-reviewed this manuscript with one of the reviewers who provided the listed reports. This is part of the Nature Communications initiative to facilitate training in peer review and to provide appropriate recognition for Early Career Researchers who co-review manuscripts.

Reviewer #3

(Remarks to the Author)
This is a beautifully executed study that demonstrates how modern biocatalysis can be applied to the de novo synthesis of lipid membranes. The enzymatic generation of cysteine-based amphiphiles and their assembly into functional, peptide-containing vesicles is technically sound and elegantly presented. The system convincingly illustrates how artificial lipid chemistries can be designed and integrated into life-like membrane constructs.

However, I find the framing of the work in a prebiotic chemistry or origins-of-life context problematic. The catalysts employed are enzymes, and the resulting lipids bear little resemblance to any prebiotically plausible or biologically abundant amphiphiles. As such, the prebiotic narrative does not add credibility; it risks distracting from the study's genuine strength, which lies in its synthetic and biocatalytic innovation.
In my opinion, this work would be most impactful if positioned squarely as a contribution to chemical biology or synthetic membrane engineering. Framed that way, the results would stand firmly on their own merit as an elegant example of artificial lipid synthesis and functional membrane construction, without invoking a prebiotic angle that the data do not substantiate. As currently framed, however, the work does not, in my view, reach the level of conceptual advance or broad impact expected for Nature Communications.

Reviewer #4

(Remarks to the Author)
Manuscript by Khanal and colleagues entitled "Breaking the membrane heredity paradox through de novo protocell formation.
This is well-executed beautiful study on the de novo synthesis of lipid membranes from small molecule precursors and water-soluble enzymes. The do novo protocell formation is challenging, important and timely, and a series of elegant experiments are performed. The research is well presented in an easy to read paper. A general point would be the possibility to synthesize unsaturated lipids and form bilayers with a lower phase transition temperature, which may have more possibilities for functional studies.

I have some suggestions and questions on the structural and functional characterization of the vesicles.
1. What are the quantities of vesicles formed and what is the polydispersity/variation in sizes and structures (both in the absence and presence of cholesterol). In most cases one (cryo-EM) or a few vesicles (phase contrast microscopy) are shown and analyzed. Some quantification of the vesicles formed (distribution of vesicle sizes) is desirable.
2. Does the synthesis of palmitoyl-CoA lead to micelle formation, from which lipid 4 is formed? What is the CMC of palmitoyl-CoA? What is the aggregation state of compound 3 at pH 8.5? Was there a particular reason to perform the experiments at pH 8.5?
3. How are the vesicles formed? Do micelles of palmitoyl-CoA aggregate and form bilayers, where then lipid 4 is synthesized? Or is lipid 4 synthesized in solution and then aggregating to form bilayers. What are the solubilities of palmitoyl-CoA and lipid 4 (and other derivatives)? Is it possible to monitor the synthesis and assembly process by light scatter measurements or other means?
4. How are the protocells growing? The experiments are done at 37 oC while the phase transition temperature is 60 oC. This suggests that the lipids are inserted into a membrane in the gel state (without cholesterol) or in a highly liquid-ordered state (with cholesterol), which I find intriguing. Is it possible to present a model of the vesicle formation and growth supported by some biophysical data?
5. I find a bilayer thickness of 2.4 nm extremely small for a dipalmitoyl lipid. The 2.4 nm value suggests a structure different from a bilayer, which would be 4-5 nm for a C16 saturated lipid.
6. The concentration of melittin (10 mol%) is extremely high, and figure S11 suggests to me that melittin destroys the vesicles rather than forming discrete pores. I am also wondering if melittin is capable of forming pores in membranes composed of saturated lipids. Did they compare the compound 4 vesicles with DPPC vesicles (with and without cholesterol)? Some data are very qualitative and based on images of one or a few vesicles (e.g. figure S13).
7. Would it be possible to characterize the vesicles quantitatively by e.g. simple calcein (de)quenching assays and determine the fraction that is permeable through melittin pore formation or lysing due to the high concentration of melittin?
8. Lines 222-224: I do not understand why membranes composed of lipids with acidic headgroups have difficulty maintaining proton gradients. Membranes composed of phosphatidylglycerol lipids are perfectly capable of doing so. I would not expect significant flip-flop of two-tail lipids on the timescale of the measurements. Is it possible that the proton permeability of lipid 4 vesicles is caused by the presence of palmitoyl-CoA or short- or long-chain fatty acids? Nevertheless, the maintenance of a significant pH gradient for 2h (figure 4g) is impressive. Would it be possible to calibrate the fluorescence signal (how much of the 4 pH unit gradient is dissipated after 2 and 12h)?
9. What determines the size of the lipids in this simple model system? Are only C16 lipids formed? What is the fraction of other chains?
10. Figure 4i shows invaginated vesicles (visible in the brightfield and fluorescence images). Invagination of vesicles occurs when the external medium has a higher osmolality than the internal medium. Such structures are less apparent in the lipid 4 vesicles of figure 4h. Do the authors have an explanation? Could they present a larger number of vesicles?

Version 1:

Reviewer comments:

Reviewer #1

(Remarks to the Author)
I have reviewed the manuscript and the authors have addressed my previous concerns.
Thanks.

Reviewer #2

(Remarks to the Author)
I co-reviewed this manuscript with one of the reviewers who provided the listed reports. This is part of the Nature Communications initiative to facilitate training in peer review and to provide appropriate recognition for Early Career Researchers who co-review manuscripts.

Reviewer #3

(Remarks to the Author)
I appreciate the effort made by the authors to respond and address reviewers' comments. I believe the new version of the manuscript, including the reframing and additional experiments requested by all reviewers, is suitable for publication in this journal.

Reviewer #4

(Remarks to the Author)
The authors have written an excellent rebuttal and revised the manuscript accordingly. I am more than satisfied with the revision.

Open Access This Peer Review File is licensed under a Creative Commons Attribution 4.0 International License, which permits use, sharing, adaptation, distribution and reproduction in any medium or format, as long as you give appropriate credit to the original author(s) and the source, provide a link to the Creative Commons license, and indicate if changes were made.
In cases where reviewers are anonymous, credit should be given to 'Anonymous Referee' and the source.
The images or other third party material in this Peer Review File are included in the article’s Creative Commons license, unless indicated otherwise in a credit line to the material. If material is not included in the article’s Creative Commons license and your intended use is not permitted by statutory regulation or exceeds the permitted use, you will need to obtain permission directly from the copyright holder.
To view a copy of this license, visit https://creativecommons.org/licenses/by/4.0/

The point-by-point responses to each comment are provided below in blue. All the changes made to the text, throughout this response letter, the revised manuscript, and the supplementary information are highlighted in yellow.

Reviewer #1

This article used protein enzymes to make palmityl-coA and then diacyl lipid from aqueous precursors, which is novel. Reactants are prebiotic using acetate and cysteine. The yields were good. The resulting protocells were then characterized for growth both with and without a general membrane pore (mellettin). The ability of these membranes to maintain a proton gradient was also determined. The paper was straightforward and of interest to a wide audience, including syth bio, OOL, astrobio, and metabolic studies.

The approach was appropriate for the goals of the paper. They used multiple types of microscopy to confirm the morphology of their structures, and the encapsulation ability of the protocells. The kinetics were also measured in more than one way, and the paper presented very clear statistical analysis in figures and figure captions.

[Authors reply] We appreciate the positive comments from the reviewer. We have carefully considered and addressed the concerns raised, as detailed below.

While the reactants were obliviously relevant to the goals of origins of life, the use of extant enzymes was not justified in the introduction. A more nuanced discussion of the role of highly evolved and cherry-picked enzymes/cofactors is warranted, especially if the authors want to claim this is important for evolution.

[Authors reply] We thank the reviewer for this comment. We agree that this point may have caused some confusion and have revised the manuscript to clarify it. Our goal was not to use modern enzymes as model prebiotic catalysts. Instead, extant enzymes are employed as convenient sources of soluble catalytic activity to test whether membrane-forming diacyl lipids can arise de novo from soluble metabolic chemistry without requiring pre-existing membranes or membrane-bound biosynthetic machinery. To make this clearer, we have revised the manuscript as follows.

(*original manuscript*)
To form lipid membranes de novo from non-lipidic metabolites, the long-chain hydrocarbon tails that are essential for membrane assembly must be synthesized from structurally simple, soluble molecules.

(*revised manuscript*)
To form lipid membranes de novo from non-lipidic metabolites, the long-chain hydrocarbon tails that are essential for membrane assembly must be synthesized from structurally simple, soluble molecules. For this purpose, we selected well-characterized enzymes that function as soluble catalysts, providing a convenient experimental platform for generating amphiphilic lipid precursors in solution.

Also Lines 38-40 state there is a gap in forming these amphiphiles, however there are lots of mechanisms for generating membrane forming materials abiotically (as demonstrated in meteorite samples, Deamer 1985) and therefore the need for this process could be late evolutionarily.
(1)

Deamer, D. W. Boundary Structures Are Formed by Organic-Components of the Murchison Carbonaceous Chondrite. Nature 1985, 317 (6040), 792–794.

[Authors reply] We thank the reviewer for this helpful comment and apologize for the lack of clarity in the original wording. Our intention was not to frame this work as addressing a limitation in abiotic amphiphile formation or as a prebiotic synthetic route. Rather, we sought to emphasize the challenge of whether lipid membranes can emerge from soluble starting materials through chemoenzymatic synthesis and be subsequently sustained by continuous lipid production. We have revised lines 38-40 of the original manuscript to clarify this point.

(*original manuscript*)
The spontaneous formation of membranes from primordial broths that supported primitive biochemistry has long been proposed[9], but no experiments have yet led to the generation of lipid membranes purely from structurally simple metabolic precursors.

(*revised manuscript*)
Although membrane-like structures have been observed in complex abiotic organic mixtures[9], lipid membrane formation from simple soluble precursors coupled to sustained lipid synthesis remains an open challenge.

Minor comments:
Positive controls for comparing growth in phospholipid membranes or proton permeation in phospholipid membranes would have been a good benchmark. (or comparison to literature values?)

[Authors reply] We thank the reviewer for this helpful suggestion. Membrane growth requires sufficient net lipid production to generate a measurable increase in vesicle surface area. In our work, although we observe significant lipid synthesis occurring, the total amount of de novo synthesized lipid was not sufficient to produce directly observable increases in vesicle size under our imaging conditions. Similar outcomes have been reported in prior studies of enzymatic phospholipid biosynthesis (*Nat. Commun.* **2020**, *11*, 4317). For this reason, we do not discuss membrane growth in the manuscript and have clarified this point in the revised text as detailed below.

(*revised manuscript*)
Consistent with prior studies, the amount of de novo synthesized lipid was insufficient to directly observe vesicle growth by optical microscopy.[11]

With regard to proton permeation, incorporation of DPPC is a common strategy in lipid membrane chemistry and drug delivery to increase bilayer packing and reduce proton permeability. DPPC bilayers have been reported to retain transmembrane pH gradients for at least six hours without detectable proton leakage (*Biomaterials*, **2012**, *33*, 3578-3585). We have expanded the discussion in the revised manuscript, compare our results with DPPC membranes, and now include the relevant reference.

(*revised manuscript*)
By comparison, the behavior of galactolipid **9** is consistent with that of tightly packed phospholipid membranes such as DPPC, which is used in liposome formulations to reduce membrane permeability and support prolonged retention of transmembrane ion and pH gradients[46].

I couldn't find the manufacturer for the Melittin.

[Authors reply] We apologize for the omission. The manufacturer of melittin has now been specified in the supplementary information.

Just an editing comment: punctuation is generally prior to ref superscripts

[Authors reply] We thank the reviewer for pointing this out. To our knowledge, the Nature family of journals specify that punctuation should follow reference superscripts, and we have therefore kept that format.

Reviewer #2

I co-reviewed this manuscript with one of the reviewers who provided the listed reports. This is part of the Nature Communications initiative to facilitate training in peer review and to provide appropriate recognition for Early Career Researchers who co-review manuscripts.

[Authors reply] We appreciate the time and effort the reviewer has dedicated to evaluating our manuscript.

Reviewer #3

This is a beautifully executed study that demonstrates how modern biocatalysis can be applied to the de novo synthesis of lipid membranes. The enzymatic generation of cysteine-based amphiphiles and their assembly into functional, peptide-containing vesicles is technically sound and elegantly presented. The system convincingly illustrates how artificial lipid chemistries can be designed and integrated into life-like membrane constructs.
However, I find the framing of the work in a prebiotic chemistry or origins-of-life context problematic. The catalysts employed are enzymes, and the resulting lipids bear little resemblance to any prebiotically plausible or biologically abundant amphiphiles. As such, the prebiotic narrative does not add credibility; it risks distracting from the study's genuine strength, which lies in its synthetic and biocatalytic innovation.
In my opinion, this work would be most impactful if positioned squarely as a contribution to chemical biology or synthetic membrane engineering. Framed that way, the results would stand firmly on their own merit as an elegant example of artificial lipid synthesis and functional membrane construction, without invoking a prebiotic angle that the data do not substantiate. As currently framed, however, the work does not, in my view, reach the level of conceptual advance or broad impact expected for Nature Communications.

[Authors reply] We thank the reviewer for the thoughtful and constructive assessment of our study and for highlighting its strengths in biocatalysis and synthetic membrane design. We appreciate the concern regarding the prebiotic framing and agree that our original presentation may have overstated this aspect. Our intention was not to present the system as a prebiotically plausible pathway or to suggest that the lipids or catalysts used here directly reflect early Earth chemistry. Rather, we sought to examine whether lipid membranes can arise de novo from completely soluble precursors in absence of pre-existing membranes and subsequently assemble into functional compartments, a question of conceptual relevance to origins of life research. In response to the reviewer's comment, we have made several revisions to the manuscript to reduce the emphasis on origin of life chemistry and to reposition the work more clearly in the context of artificial lipid synthesis, and functional membrane construction.

Specifically, we have implemented changes in the abstract, removing references to prebiotic intermediates and emphasizing the direct coupling between lipid synthesis, self-assembly, and compartment function as the principal advance. Throughout the main text, we have consistently reduced discussion of early cellular evolution and instead frame the work in terms of bottom-up membrane construction, focusing on amphiphile generation from soluble feedstocks. Statements invoking prebiotic plausibility of the proposed system have been removed or rewritten to focus on mechanistic interpretation. The conclusion has been completely rewritten and now emphasizes that the key conceptual contribution is the demonstration that membrane assembly can emerge directly from soluble reaction networks, with lipid synthesis, self-organization, and compartment function tightly coupled. Please note that the revisions in the manuscript are highlighted in yellow.

We believe these revisions clarify the scope of the study and more accurately position it as an advance in artificial lipid synthesis and functional membrane construction.

Reviewer #4

Manuscript by Khanal and colleagues entitled "Breaking the membrane heredity paradox through de novo protocell formation.
This is well-executed beautiful study on the de novo synthesis of lipid membranes from small molecule precursors and water-soluble enzymes. The do novo protocell formation is challenging, important and timely, and a series of elegant experiments are performed. The research is well presented in an easy to read paper.

[Authors reply] We appreciate the positive comments from the reviewer. We have carefully considered and addressed the concerns raised, as detailed below.

A general point would be the possibility to synthesize unsaturated lipids and form bilayers with a lower phase transition temperature, which may have more possibilities for functional studies.

[Authors reply] We thank the reviewer for this thoughtful suggestion. We considered the possibility of introducing unsaturated lipids by incorporating desaturase enzymes. However, the majority of membrane lipid desaturases are membrane-bound or membrane-associated enzymes, often operating on lipids that are already incorporated into a membrane, making them difficult to implement under the membrane-free conditions central to our system. For this reason, implementing enzymatic desaturation within the current framework is not straightforward. We nevertheless agree that controlling lipid unsaturation represents an important direction for expanding the functional scope of these assemblies. Accordingly, we have added a statement in the conclusion highlighting the development of strategies to introduce unsaturation into de novo generated synthetic membranes as a promising future direction.

(*revised manuscript*)
Controlling lipid unsaturation will be an important direction for future work, particularly for accessing bilayers with lower phase transition temperatures that better support functional studies.

I have some suggestions and questions on the structural and functional characterization of the vesicles.

1. What are the quantities of vesicles formed and what is the polydispersity/variation in sizes and structures (both in the absence and presence of cholesterol). In most cases one (cryo-EM) or a few vesicles (phase contrast microscopy) are shown and analyzed. Some quantification of the vesicles formed (distribution of vesicle sizes) is desirable.

[Authors reply] We thank the reviewer for this comment. In response, we performed DLS analysis on the de novo membrane formation samples and observed high polydispersity both in the absence and presence of cholesterol. This is consistent with our imaging data, as vesicles were detected by both cryo-EM and optical microscopy, indicating the presence of structures spanning nanometer and micrometer length scales. This behavior is also consistent with our previous work on de novo membrane formation, where DLS likewise showed highly polydisperse samples with vesicles ranging from nanometer to micrometer sizes after in situ lipid formation (*Angew. Chem Int. Ed.* **2023**, e202311635; *Angew. Chem Int. Ed.* **2022**, e202200549). We have added this clarification to the revised manuscript.

(*revised manuscript*)
To further characterize the vesicle size distribution upon in situ formation of lipid **4**, we carried out dynamic light scattering (DLS) analyses in the absence and presence of cholesterol (Supplementary Fig. 8). In both conditions, the DLS profiles revealed highly polydisperse samples, consistent with the vesicle population spanning nanometer to micrometer length scales, in agreement with previous findings[36-37].

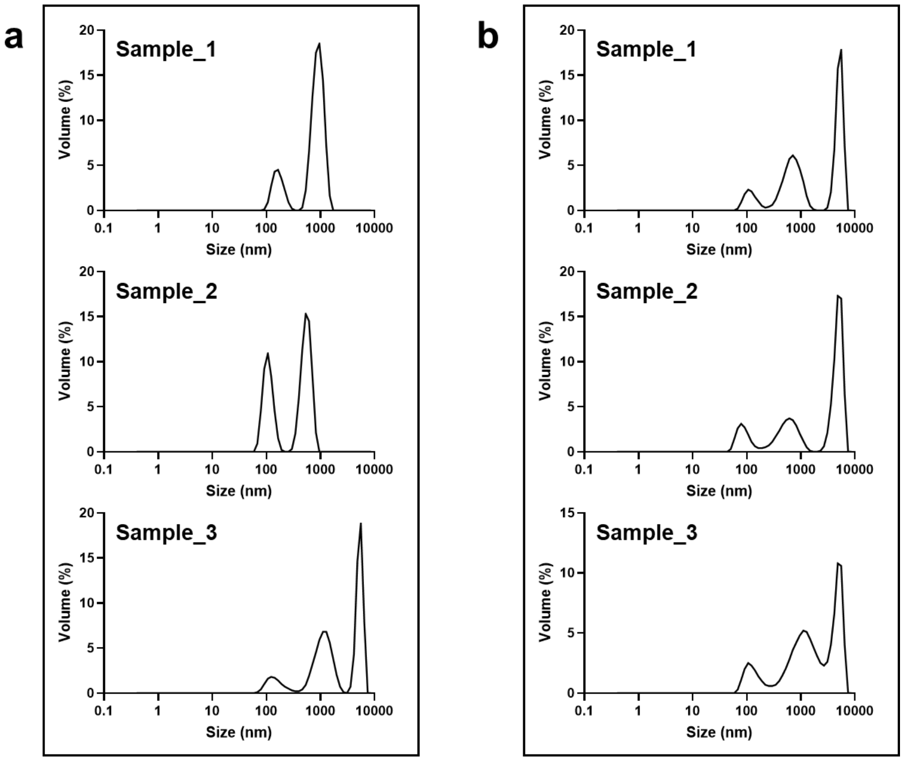

Supplementary Fig. 8. DLS analyses showing size distribution of in situ formed vesicles of lipid **4** **(a)** in absence of cholesterol (PDI values: Sample_1 = 0.83, Sample_2 = 0.67, Sample_3 = 0.81), and **(b)** in presence of 10 mol% cholesterol (PDI values: Sample_1 = 0.45, Sample_2 = 0.47, Sample_3 = 0.50).

2. Does the synthesis of palmitoyl-CoA lead to micelle formation, from which lipid 4 is formed? What is the CMC of palmitoyl-CoA? What is the aggregation state of compound 3 at pH 8.5? Was there a particular reason to perform the experiments at pH 8.5?

[Authors reply] We thank the reviewer for these important questions. Palmitoyl-CoA forms micelles at concentrations well below those obtained in our chemoenzymatic approach, with a reported critical micelle concentration (cmc) of about 35 µM at pH 8.5 (*J. Colloid Interface Sci.* **1993**, *161*, 343-346). Reported values decrease further with increasing ionic strength and change little between neutral and mildly basic pH. Since in our system the enzymatic formation of palmitoyl-CoA is considerably faster than the subsequent chemical diacylation of cysteine, we expect palmitoyl-CoA to first accumulate as micellar aggregates from which lipid **4** is formed. The observation of only trace amounts of monoacylated intermediate is consistent with this interpretation and aligns with our previous observations (*Nat. Chem.* **2025**, *17*, 148-155; *Angew. Chem. Int. Ed.* **2025**, *64*, e202421932) that aggregation of amphiphilic thioesters can promote rapid sequential acylation by maintaining reactants in close proximity. Also, please refer to the manuscript revisions below and the additional response to comment 3.

The reactions were carried out at pH 8.5 because this condition supports both efficient enzymatic lipid tail synthesis and rapid chemical acylation. In particular, pH 8.5 is close to the reported pK_a of the free cysteine thiol (8.3-8.5) (*Biochemistry* **2008**, *47*, 12860-12868), so a substantial fraction of cysteine is present as the thiolate nucleophile required for the acylation step. At the same time, the ACS, ACC, and FAS enzymes used retain robust activity under mildly basic conditions.

We have clarified these points in the revised manuscript as detailed below.

(*revised manuscript*)
A pH of 8.5 was selected to increase the fraction of cysteine present as thiolate, facilitating the first acylation step[30]. We confirmed synthesis of **4** by comparison with a chemically synthesized standard (Supplementary Fig. 5). After 8 hours, we observed 1.6±0.3 mM of lipid **4**, corresponding to 80% yield. Intriguingly, we observed only trace formation of the monoacylated lipid product, including at early time points. Even when using substoichiometric amounts of palmitoyl-CoA, we primarily observed formation of the diacylated compound **4** with minimal formation of monoacylated cysteine (Supplementary Fig. 5). Considering the low critical micelle concentration (cmc) reported for palmitoyl-CoA (35 µM at pH 8.5)[31], we attribute our observation to the rate-limiting step being the first acylation. Once acylated, the lipid likely forms mixed micelles with palmitoyl-CoA, positioning another thioester in proximity to the reactive thiol, leading to a more rapid second acylation.

3. How are the vesicles formed? Do micelles of palmitoyl-CoA aggregate and form bilayers, where then lipid 4 is synthesized? Or is lipid 4 synthesized in solution and then aggregating to form bilayers. What are the solubilities of palmitoyl-CoA and lipid 4 (and other derivatives)? Is it possible to monitor the synthesis and assembly process by light scatter measurements or other means?

[Authors reply] We thank the reviewer for this insightful question. Elucidating how transitions between different supramolecular assemblies occur during lipid synthesis is an important and biologically relevant direction that we are actively pursuing in simpler lipid membrane systems. In general, following these transitions by light scattering is challenging, because larger particles dominate the scattering signal and mask contributions from smaller assemblies, limiting mechanistic interpretation. We are currently working on establishing time-resolved cryo-EM

approaches in simpler and more tractable model reaction systems and expect these methods to provide a more direct means to address this question in future work, but we consider these future studies beyond the scope of the present study.

Our evidence suggests that the reaction begins within palmitoyl-CoA micelles rather than in bulk solution. Palmitoyl-CoA is known to form micelles under the conditions used in this work, and the trace amount of monoacylated intermediate observed supports a mechanism in which sequential acylation occurs within preformed aggregates. As lipid **4** accumulates, micelles transition toward bilayer structures. Considering that dipalmitoylphosphatidylcholine (16:0 PC) has a cmc of 0.46 nM and that the cysteine headgroup is less polar than the phosphocholine headgroup, lipid **4** is expected to have a comparable or lower cmc.

We have clarified these points in the revised manuscript as detailed below.

(*revised manuscript*)
Since the formation of palmitoyl-CoA **3** is considerably faster than the subsequent chemical diacylation of cysteine, we propose that vesicle formation may proceed through initial formation of compound **3** micelles, within which lipid **4** is generated, followed by transition to bilayer structures.

4. How are the protocells growing? The experiments are done at 37 oC while the phase transition temperature is 60 oC. This suggests that the lipids are inserted into a membrane in the gel state (without cholesterol) or in a highly liquid-ordered state (with cholesterol), which I find intriguing. Is it possible to present a model of the vesicle formation and growth supported by some biophysical data?

[Authors reply] We thank the reviewer for this insightful comment. Our working model is that ongoing lipid synthesis does not proceed by insertion of the final diacyl lipid **4** into pre-existing membranes, but rather by intercalation of the single-chain amphiphile palmitoyl-CoA into bilayer assemblies, followed by its conversion to lipid **4**. This remains feasible even below the phase transition temperature of lipid **4**, as the insertion of single-chain amphiphiles is expected to be more favorable than insertion of diacyl lipids. Consistent with this model, generalized polarization (GP) measurements of hydrated lipid **4** vesicles showed positive values indicative of highly ordered membranes, whereas addition of palmitoyl-CoA caused a marked decrease in GP, supporting its intercalation into pre-existing bilayers. We have revised the manuscript as follows.

(*revised manuscript*)
Once bilayers are formed, expansion can proceed through transfer of palmitoyl-CoA from micelles into pre-existing membranes, where tighter packing might promote retention relative to micelles, followed by conversion of palmitoyl-CoA to lipid **4**. Consistent with this mechanism, generalized polarization (GP) measurements show a pronounced decrease upon addition of palmitoyl-CoA to hydrated vesicles of lipid **4**, suggesting intercalation into pre-existing membranes even when starting from fully formed vesicles (Supplementary Fig. 10).

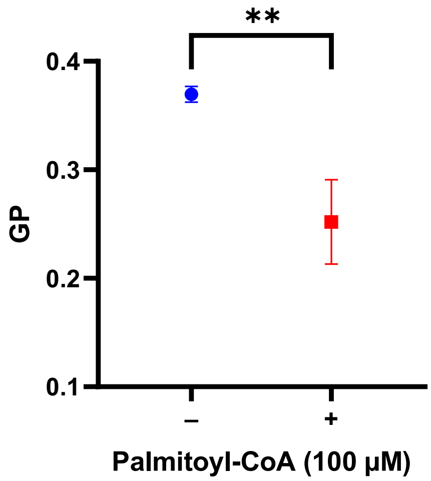

Supplementary Fig. 10. Laurdan GP values of hydrated vesicles of lipid **4** in absence (blue) and presence (red) of palmitoyl-CoA. Data points presented as means ± s.d. (n = 3 technically independent samples), and the significance determined using an unpaired t-test (two tailed). (–) vs (+) palmitoyl-CoA **P = 0.0067.

5. I find a bilayer thickness of 2.4 nm extremely small for a dipalmitoyl lipid. The 2.4 nm value suggests a structure different from a bilayer, which would be 4-5 nm for a C16 saturated lipid.

[Authors reply] We apologize for the confusion caused by our initial analysis. The originally reported value (2.4 nm) underestimated the bilayer thickness because it was calculated from the distance between the minima of the radial intensity profile, following a simplified implementation of the radial profiling approach described in *Proc. Natl. Acad. Sci.* **2020**, *117*, 19943–19952. We recognized that this metric does not capture the full bilayer signal. We have therefore corrected the analysis and now define bilayer thickness as the full width of the dark-bright-dark feature in the averaged radial profile. This yields a value of ~5 nm, consistent with expected values for saturated C16 bilayers.

6. The concentration of melittin (10 mol%) is extremely high, and figure S11 suggests to me that melittin destroys the vesicles rather than forming discrete pores. I am also wondering if melittin is capable of forming pores in membranes composed of saturated lipids. Did they compare the compound 4 vesicles with DPPC vesicles (with and without cholesterol)? Some data are very qualitative and based on images of one or a few vesicles (e.g. figure S13).

[Authors reply] We thank the reviewer for this comment. We agree that 10 mol% melittin is a relatively high loading, but similar concentration ranges have been commonly reported in the literature. In particular, previous studies have shown that melittin is predominantly surface bound at low peptide to lipid ratios, reorients above about 4 mol%, and adopts a pseudotransmembrane α helical structure at 9 mol%, including in saturated lipid bilayers such as DPPC (*Biophys. J.* **2005**, *89*, 3214-3222).

We apologize for the confusion caused by Figure S11 and have revised the figure and caption accordingly (now supplementary Fig. 13). Figure S11c and d show the same sample in presence of melittin in two fluorescence channels. Figure S11c shows that vesicles are still present in the dispersion, as the membrane is stained with Texas Red DHPE. Figure S11d shows that the hydrophilic dye HPTS is no longer confined within the vesicles and is instead distributed inside and outside, consistent with leakage.

A direct comparison between pore formation in DPPC vesicles and our system is not straightforward because our vesicles are chemoenzymatically generated and contain multiple components, rather than a simple phospholipid or phospholipid cholesterol mixture.

Finally, we have replaced the original Figure S13a, which showed a single vesicle, with a new image that includes multiple vesicles (Supplementary Fig. 15a).

(*original Figure S11*)

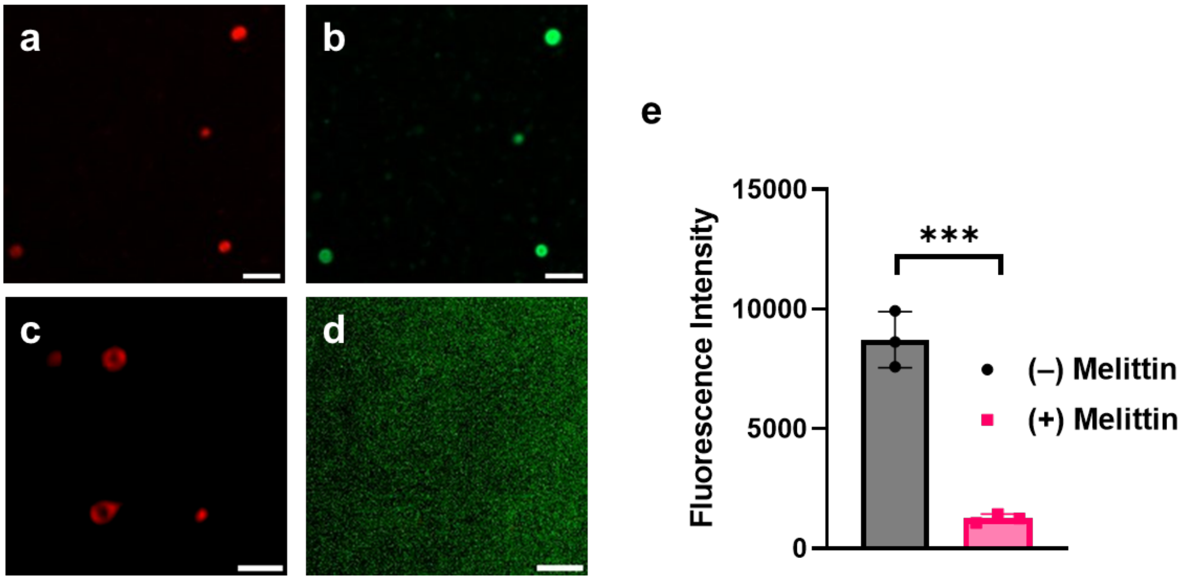

(*revised Supplementary Fig. 13*)

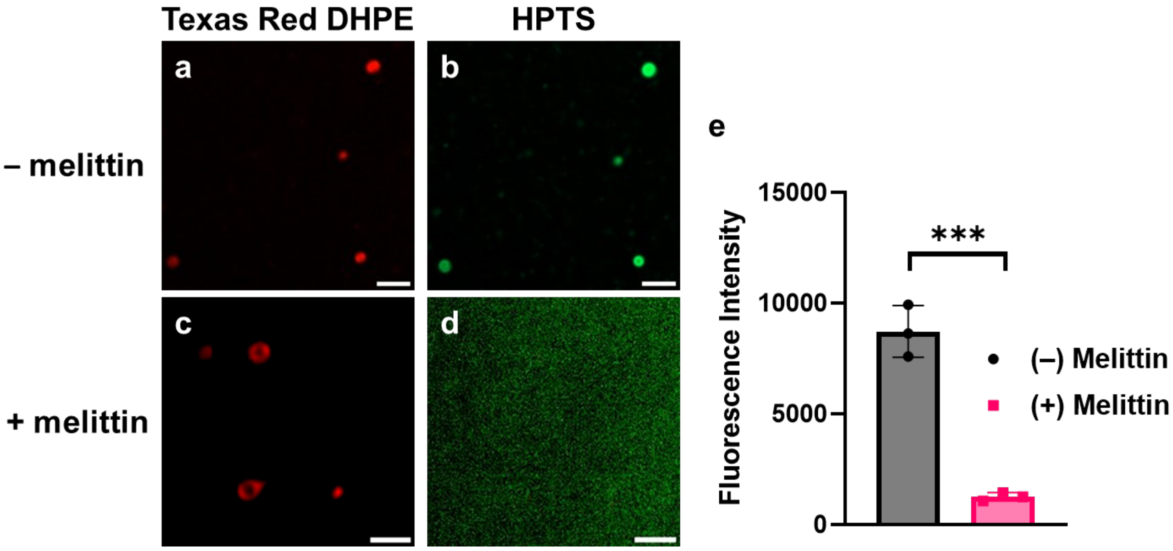

(*original Figure S13*)

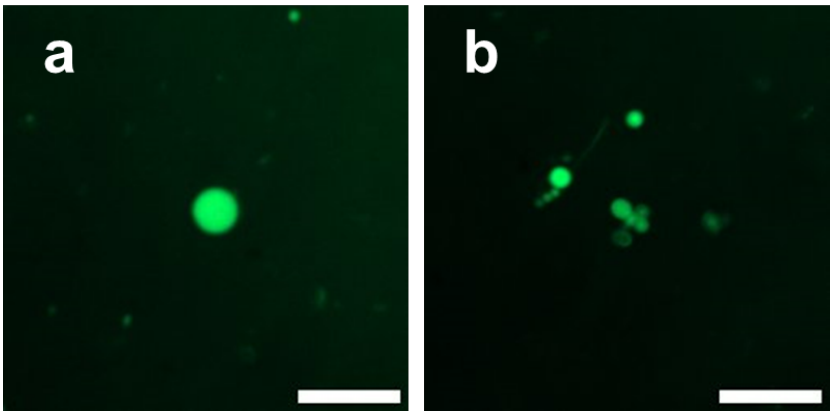

(*revised Supplementary Fig. 15*)

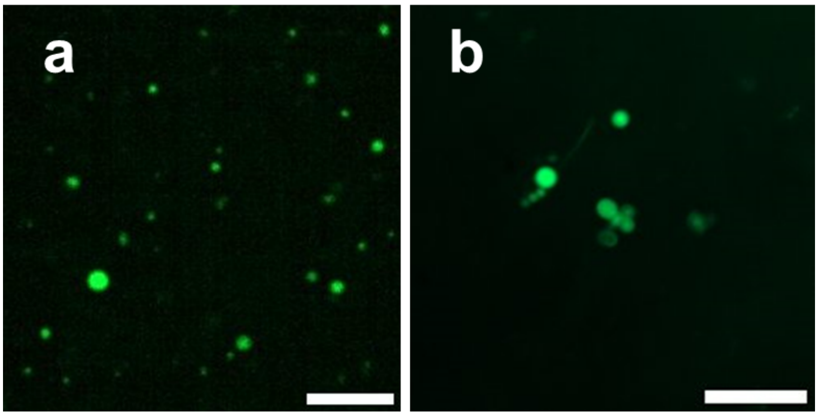

7. Would it be possible to characterize the vesicles quantitatively by e.g. simple calcein (de)quenching assays and determine the fraction that is permeable through melittin pore formation or lysing due to the high concentration of melittin?

[Authors reply] We thank the reviewer for this suggestion. In principle, a calcein dequenching assay could provide an ensemble averaged readout of cargo release. However, in our system this experiment is not straightforward, because it would require encapsulation of calcein at high self-quenched concentrations during the chemoenzymatic vesicle generation process, and we cannot exclude that these conditions would interfere with vesicle formation. In addition, the assay would report overall loss of compartmentalization and would not allow a reliable distinction between pore mediated leakage and vesicle rupture. Since the main concern here is vesicle rupture, we refer to our response to Comment 6, where we clarify that vesicles remain present under these conditions.

8. Lines 222-224: I do not understand why membranes composed of lipids with acidic headgroups have difficulty maintaining proton gradients. Membranes composed of phosphatidylglycerol lipids are perfectly capable of doing so. I would not expect significant flip-flop of two-tail lipids on the timescale of the measurements. Is it possible that the proton permeability of lipid 4 vesicles is caused by the presence of palmitoyl-CoA or short- or long-chain fatty acids? Nevertheless, the maintenance of a significant pH gradient for 2h (figure 4g) is impressive. Would it be possible to calibrate the fluorescence signal (how much of the 4 pH unit gradient is dissipated after 2 and 12h)?

[Authors reply] We thank the reviewer for this comment. We agree that our original wording was too broad. We did not intend to imply that membranes containing acidic or negatively charged phospholipids are generally unable to maintain proton gradients. Our rationale instead concerns lipids bearing a carboxylic acid headgroup and not a phosphate. Fatty acid vesicles are well known to dissipate proton gradients because the protonated neutral form can traverse the membrane and release the proton on the opposite side. We therefore expected the diacylated Cys amphiphile **4** to display related behavior, although likely with slower transbilayer migration than a single chain fatty acid because of the second hydrocarbon tail. This interpretation is consistent with our experimental observations. This reduced transbilayer mobility represents an important advantage over fatty acid vesicles, and we appreciate the reviewer's comment for prompting us to clarify this point in the manuscript.

This reasoning does not apply in the same way to acidic phospholipids such as PA or PG, because these headgroups are substantially more acidic and therefore remain predominantly charged. For comparison, PA has a reported first pKa of about 3 (*Biochemistry* **2005**, *44*, 17007-17015), whereas fatty acids have a pKa of about 4.8 in water when free in water, and in vesicle assemblies their apparent pKa can increase to about 7.5. Furthermore, we consider residual palmitoyl-CoA unlikely to account for the proton permeability, because vesicles formed from the galacto-HG amphiphile are prepared under identical conditions and would be expected to contain comparable or higher amounts of residual palmitoyl-CoA, yet they retain the proton gradient. This indicates that the observed proton permeability is specific to lipid **4** rather than arising from residual starting material.

9. What determines the size of the lipids in this simple model system? Are only C16 lipids formed? What is the fraction of other chains?

[Authors reply] We thank the reviewer for this question. Based on our previous work (*J. Am. Chem. Soc.* **2021**, 143, *23*, 8533-8537), we chose type I FAS B from *Corynebacterium glutamicum* (cgFAS1B) because it is reported to lead to the primary formation of palmitoyl-CoA (*Microbiology* **2005**, *151*, 2421-2427). As described in our previous work, cgFAS1B primarily produces palmitoyl-CoA, and the lipids formed in situ are therefore expected to be dominated by C16 species. Although minor amounts of other chain lengths below the detection limit cannot be excluded, we did not observe additional lipid products in the HPLC profile under our experimental conditions.

10. Figure 4i shows invaginated vesicles (visible in the brightfield and fluorescence images). Invagination of vesicles occurs when the external medium has a higher osmolality than the internal medium. Such structures are less apparent in the lipid 4 vesicles of figure 4h. Do the authors have an explanation? Could they present a larger number of vesicles?

[Authors reply] We thank the reviewer for this observation. The difference was due to the particular images selected for the original figure, rather than to a systematic difference between the samples. In the revised manuscript, we have added a larger number of representative vesicle images in the supporting information so that the data presentation better reflects the overall population.

(*revised Supplementary Fig. 19*)

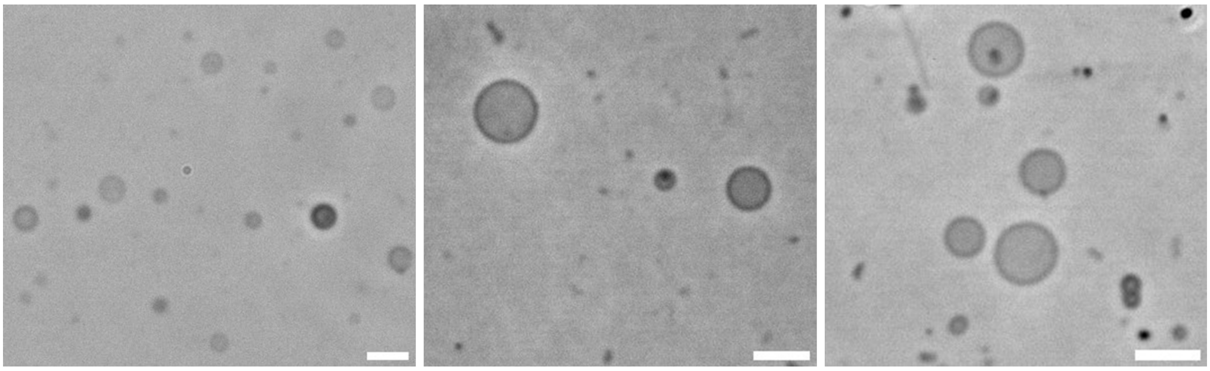

Supplementary Fig. 19. Phase-contrast microscopy images of vesicles of galactolipid **9** formed by dipalmitoylation of galacto-HG **8** using palmitoyl-CoA in the presence of 10 mol% cholesterol in bicine buffer (pH 8.5), acquired after external buffer exchange with citrate buffer (pH 4.6). Scale bar = 5 µm.
